# Impact of iodine nutritional levels in urban and rural pregnant women on neonatal growth indicators: a cohort study in Xinjiang, China

**DOI:** 10.3389/fnut.2025.1668818

**Published:** 2025-10-01

**Authors:** Jing Zhou, Rishalaiti Tayier, Dannier Abuduwaili, Dawureni Muhetaer, Chenchen Wang, Kai Pan

**Affiliations:** ^1^School of Public Health, Xinjiang Medical University, Urumqi, China; ^2^Health Hazard Factor Detection and Control Institute, Xinjiang Uygur Autonomous Region Center for Disease Control and Prevention, Urumqi, China

**Keywords:** iodine nutrition, pregnant women, newborns, body length, serum iodine

## Abstract

**Objectives:**

Iodine nutrition during pregnancy is crucial for infant health. This study aimed to investigate regional heterogeneity in iodine nutrition levels among urban and rural/agro-pastoral women and its association with neonatal growth indicators, providing evidence for region-specific nutritional interventions.

**Methods:**

The study enrolled 85 urban pregnant women from Urumqi City and 181 rural/agro-pastoral pregnant women from the Ili Kazakh Autonomous Prefecture. Basic demographic data were collected via questionnaires, and maternal serum and random urinary iodine concentrations were determined using whole blood and urine samples collected during early to mid-pregnancy. After balancing baseline differences between urban and rural groups using inverse probability weighting, the relationship between maternal iodine nutrition and neonatal birth outcomes, as well as its regional interaction effects, were analyzed.

**Results:**

The median serum iodine levels in urban and rural/agro-pastoral pregnant women were 84.44 (75.50, 97.10) μg/L and 246.41 (186.33, 322.54) μg/L, respectively, showing a significant difference (*p* < 0.05). Regional interactions were observed between maternal serum/urinary iodine levels and neonatal birth length. In rural/agro-pastoral regions, iodine nutrition exhibited a nonlinear association with neonatal length (optimal serum iodine range: 100.62–254.20 μg/L; optimal random urinary iodine range: 106.16–210.80 μg/L). In contrast, urban pregnant women mostly displayed linear or nonsignificant associations between iodine levels and neonatal growth indicators.

**Conclusion:**

The impact of maternal iodine nutrition on neonatal growth differs between urban and rural/agro-pastoral areas. A nonlinear association with an optimal range was observed between iodine nutrition and neonatal length in rural/agro-pastoral regions. These findings provide a basis for developing region-specific iodine nutrition intervention strategies. It should be noted, however, that the urban group had a relatively small sample size, and the statistical power for between-group comparisons was limited. Therefore, the conclusions warrant further validation.

## 1 Introduction

Iodine, as an essential trace element, plays an irreplaceable role in the synthesis and secretion of thyroid hormones (1). Thyroid hormones are critical for fetal neurological development, skeletal growth, and the differentiation and maturation of various organ systems. Maternal iodine nutritional status during pregnancy directly impacts fetal growth and long-term health outcomes (2, 3). Studies demonstrate that iodine deficiency in pregnant women can lead to fetal brain developmental disorders, increasing the risk of congenital iodine deficiency syndrome, intellectual disability, and congenital hypothyroidism. Conversely, excessive iodine intake may also disrupt thyroid hormone synthesis and metabolism, adversely affecting fetal thyroid function (4–6).

The World Health Organization (WHO), based on global iodine nutrition surveys, recommends an optimal urinary iodine concentration range of 150–249 μg/L for pregnant women, establishing a unified benchmark for global maternal iodine nutrition management (7). However, this criterion may not be fully applicable to the Xinjiang region of China. Located in the inland northwest of China, Xinjiang is characterized by unique geological conditions that result in an iodine-deficient environment, making it a historically endemic area for iodine deficiency disorders (IDD) (8, 9). Since the implementation of the universal salt iodization (USI) policy in China in 1995, measures such as subsidized iodized salt and iodine supplementation for high-risk populations have effectively controlled the prevalence of IDD to some extent. Nevertheless, due to geographical, economic, and dietary cultural differences, iodine nutrition status remains uneven across different areas of Xinjiang (10). As the provincial capital, Urumqi exhibits dietary patterns influenced considerably by modern lifestyles, whereas pastoral and agricultural regions such as the Ili Kazakh Autonomous Prefecture rely primarily on traditional animal husbandry and farming, with minimal seafood consumption. Moreover, Xinjiang is a multi-ethnic region, and variations in dietary habits and food composition among different ethnic groups further complicate the iodine nutrition status of pregnant women (11). Previous studies have indicated regional disparities in iodine content in soil and water sources across parts of Xinjiang (12, 13). These unique environmental factors may lead to differences in iodine intake among pregnant women compared to other regions (14). Therefore, conducting research on the relationship between iodine nutrition in pregnant women and neonatal growth and development in Xinjiang carries profound regional significance and scientific value.

Although existing studies have focused on the effectiveness of prevention and control strategies for iodine deficiency disorders in Xinjiang, most remain limited to descriptive analyses of the current situation (15, 16). Previous research has yet to provide systematic evidence regarding how distinct dietary cultures in urban and rural areas of Xinjiang’s unique geographic environment contribute to disparities in iodine nutrition among pregnant women and the resulting heterogeneity in neonatal health outcomes (17, 18). Most importantly, it remains unclear whether the internationally recommended optimal range of iodine nutrition is applicable to populations in Xinjiang’s farming and pastoral areas, and whether there exists a region-specific safe window of iodine exposure. Therefore, this study compares pregnant women from urban Urumqi and rural Yili farming-pastoral areas in Xinjiang to untangle the distribution characteristics of iodine nutrition levels under urban–rural disparities. The findings aim to provide a scientific basis for developing iodine nutrition intervention strategies tailored to the regional characteristics of Xinjiang, and may also offer targeted support for iodine nutrition management in pregnant women and health promotion among newborns in the region.

## 2 Materials and methods

### 2.1 Study participants

The study subjects comprised urban pregnant women residing in Urumqi City and pregnant women from rural/agro-pastoral areas in the Ili Kazakh Autonomous Prefecture (all healthy women in their first or second trimester with a local residency duration of over 3 years). Initially, 100 urban and 200 rural pregnant women were enrolled. After excluding cases with incomplete data, missing samples, multiple pregnancies, preterm births, and loss to follow-up, 85 urban and 181 rural pregnant women were ultimately included. This study was approved by the Ethics Committee of the Xinjiang Uygur Autonomous Region Center for Disease Control and Prevention (2022-05) and complied with the “Ethical Review Measures for Biomedical Research Involving Human Subjects (Trial)” and the “Declaration of Helsinki.” All participating women provided written informed consent.

### 2.2 Sample collection

A questionnaire survey was conducted among pregnant women, collecting data on name, age, height, weight, education level, occupation, gestational week, expected delivery date, and personal medical history. During routine prenatal examinations, 4 mL each of whole blood and urine were collected by professional phlebotomists from the obstetric clinic. Postpartum follow-up was conducted to record neonatal information, including sex, birth length, birth weight, health status at birth, and preterm birth status.

### 2.3 Laboratory testing

After whole blood collection, samples were allowed to stand at room temperature for 30 min. Serum was separated using a TDZ4-WS benchtop low-speed centrifuge with a rotor radius of 15 cm at 3,500 rpm for 10 min. The total iodine concentration in maternal serum was determined by inductively coupled plasma mass spectrometry (ICP-MS) (19). The urinary iodine concentration in random spot urine samples from pregnant women was measured using the arsenic-cerium catalytic spectrophotometric method (20).

### 2.4 Statistical methods

To control for confounding bias in baseline characteristics between urban and rural/agro-pastoral pregnant women, inverse probability weighting (IPW) was employed to balance the distribution of covariates across groups (21). First, a logistic regression model was constructed with the regional grouping (urban = 0, rural/agro-pastoral = 1) as the dependent variable to estimate propensity scores. The specific formula is as follows:

$$\text{logit}(P(\text{region}=1|X\;))=\beta_{0}+\sum_{i=1}^{8}\beta_{i}\cdot X_{i}$$

Here, p(region = 1|X) represents the conditional probability of an individual belonging to the rural/agro-pastoral group given the covariates; β_0_ is the intercept term; *X_i_* denotes the baseline covariates to be balanced (including maternal age at initial survey, body mass index, gestational age, occupation, education level, and neonatal sex, with primary education as the reference); and β*_i_* is the regression coefficient for the corresponding covariate. Based on the propensity score P(X), inverse probability weights were calculated, and extreme weights were adjusted via oversampling parameters. Covariate balance after weighting was assessed using a standardized mean difference (SMD) threshold of <0.1 (22). Subsequent statistical analyses were conducted using the weighted balanced dataset.

Generalized additive models (GAM) were applied to examine the interactions between region and maternal iodine nutrition levels on neonatal birth outcomes (23). For the four combinations of maternal serum iodine/urinary iodine and neonatal birth length/weight, GAMs incorporating regional stratification were constructed. The specific formula is as follows:

$$\begin{array}{c} Y=\beta_{0}+\text{region}+s(\text{Iodine},\mathrm{by}=\text{region},k=4)+s(\mathrm{age},k=4)+s(\mathrm{bmi},k=4)\\ \;+\text{gestational week}+\text{educational}+\text{career}+\text{neonatal}\;\mathrm{sex}+\epsilon \end{array}$$

Here, *Y* represents neonatal birth outcomes; β_0_ is the intercept term; s(Iodine, by = region, *k* = 4) is the interaction term, where stratified smoothing curves were used to fit the nonlinear effects of iodine exposure in rural and urban groups; *k* = 4 was set to restrict degrees of freedom and prevent overfitting. Likelihood ratio tests were performed to compare models with and without the interaction term. Nonlinear relationships were verified using the effective degrees of freedom (EDF > 1.2) of the smoothing term and F-statistics (24).

Upon confirming the interaction between region and maternal iodine nutrition levels on neonatal birth outcomes, restricted cubic spline (RCS) models were employed to fit the nonlinear relationship between iodine exposure and neonatal outcomes (25). The specific formula is as follows:

$$Y=\beta_{0}+\sum_{j=1}^{4}\beta_{j}\cdot \mathrm{RCS}(\text{Iodine},\text{knots})+\sum \beta_{k}\cdot X+\epsilon$$

Here, *Y* denotes neonatal birth outcomes; β_0_ is the intercept term; β*_j_* represents the coefficients of the restricted cubic spline function; RCS(Iodine, knots) constructs a spline basis function with four knots to characterize the nonlinear association between maternal iodine nutrition levels and neonatal outcomes (26); *X* denotes covariates, and β*_k_* is the corresponding coefficient. Potential thresholds were identified by computing the first derivative (slope) and second derivative (curvature) of the curve, with inflection points determined by changes in curvature sign and derivative direction (27).

Data were processed using Excel 2021, and statistical analyses were performed in R4.3.4 with the following key packages: WeightIt, mgcv., rms, tidyverse, cobalt, and ggplot2.

## 3 Results

### 3.1 Basic characteristics of pregnant women and neonates

A total of 266 mother-infant pairs were included in this study, with detailed characteristics presented in Table 1. At the initial survey, significant differences were observed between urban and rural/agro-pastoral pregnant women in gestational age, occupation, and maternal serum iodine levels. Specifically, the median gestational age of urban pregnant women was 12 (9, 17) weeks, compared to 19 (14, 23) weeks in rural/agro-pastoral pregnant women. The proportion of unemployed urban pregnant women was 54.12%, while that of rural/agro-pastoral pregnant women was 75.69%. The median maternal serum iodine level was 84.44 (75.50, 97.10) μg/L in urban women and 246.41 (186.33, 322.54) μg/L in rural/agro-pastoral women. A statistically difference was also observed in neonatal birth length between the two regions, with urban neonates measuring 50 (50, 52) cm and rural/agro-pastoral neonates measuring 50 (50, 51) cm. However, no significant differences were found in neonatal sex or birth weight between the two groups.

**Table 1:** Basic characteristics of mother-infant pairs.

Variable	Urban (*n* = 85)	Rural/agro-pastoral (*n* = 181)	*P*-value
Maternal age (years)	28 (26, 32)	29 (26, 33)	0.374
Maternal BMI (kg/m^2^)	23.77 (21.16, 26.57)	24.98 (22.41, 27.52)	0.054
Gestational age (weeks)	12 (9, 17)	19 (14, 23)	**<0.001**
Maternal education			0.118
Primary school	7.06%	14.36%	
Junior high school	34.12%	38.12%	
Vocational/high school	16.47%	18.23%	
College or higher	42.35%	29.28%	
Maternal occupation			**<0.001**
Unemployed	54.12%	75.69%	
Employed	45.88%	24.31%	
Serum iodine (μg/L)	84.44 (75.50, 97.10)	246.41 (186.33, 322.54)	**<0.001**
Random urinary iodine (μg/L)	192.55 (12.95, 236.49)	196.23 (165.85, 232.10)	0.106
Neonatal sex			0.883
Female	48.24%	46.41%	
Male	51.76%	53.59%	
Birth length (cm)	50 (50, 52)	50 (50, 51)	**0.025**
Birth weight (g)	3,600 (3,290, 3,885)	3,480 (3,200, 3,800)	0.079

All maternal characteristics were obtained at the initial survey. Bold text indicates statistical significance.

### 3.2 Equations comparison of covariate balance before and after propensity score matching

**Figure 1:**
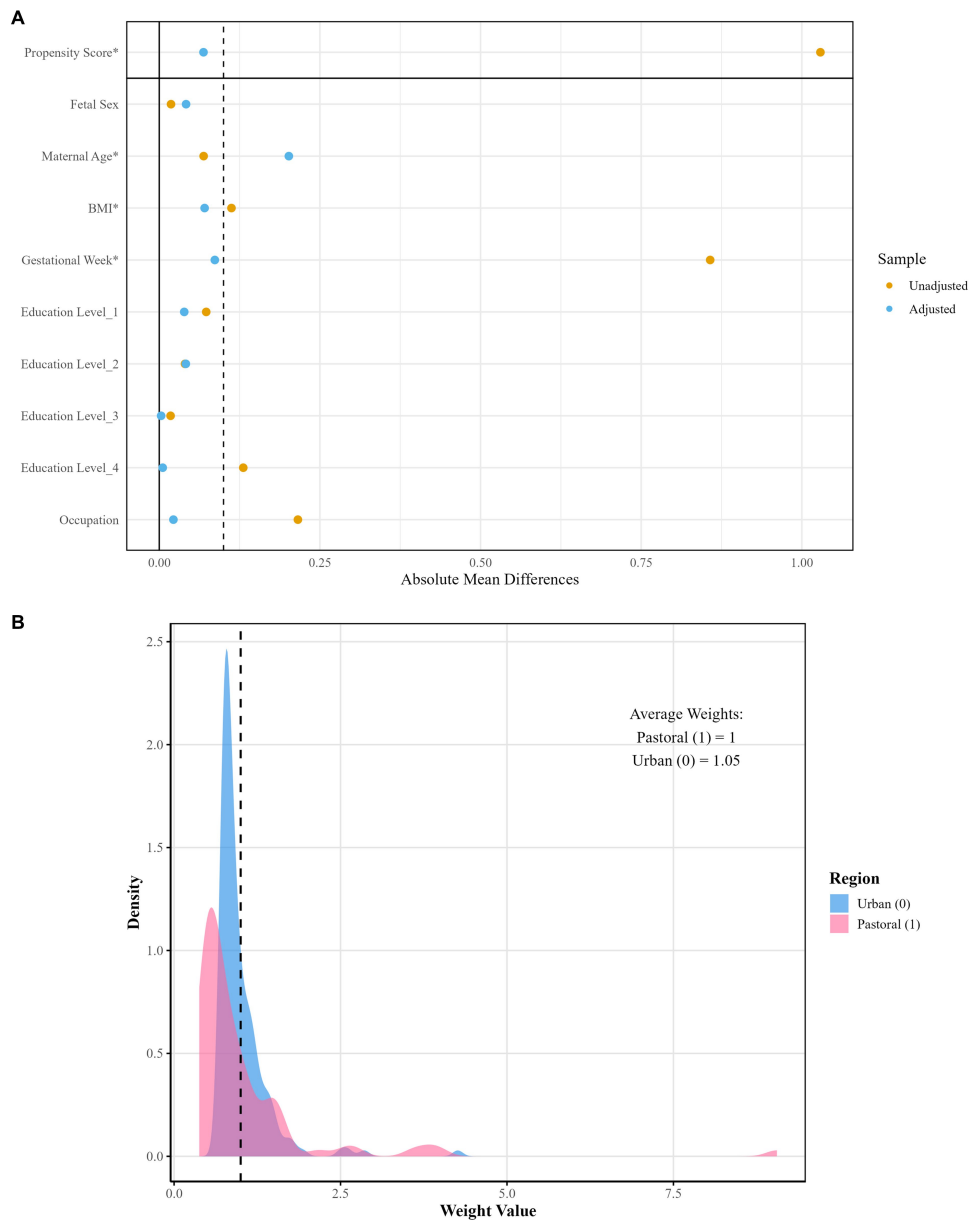
Visualization of weight distribution density and covariate balance after propensity score matching. **(A)** Illustrates covariate balance comparison; **(B)** displays weight distribution density.

After propensity score matching, the balance of covariates before and after weighting was evaluated using Table 2 and Figure 1. The results demonstrated that, except for maternal age, the absolute SMD of all other covariates were below 0.1, meeting the balance criterion. Following IPW, the average weights in the urban and rural groups were close to 1 (rural/agro-pastoral area = 1.00, urban = 1.05), indicating no substantial deviation from the equal-weight benchmark. Although the right-tailed distribution in the urban group suggested the presence of extreme weights, it did not compromise the overall balance.

**Table 2:** Comparison of standardized mean differences (SMD) before and after weighting.

Covariate	Unweighted SMD	Weighted SMD
Propensity score	1.0289	−0.069
Neonatal sex (female = 0, male = 1)	0.0183	0.042
Maternal age	0.069	−0.202
Maternal BMI	0.112	0.071
Gestational age	0.857	−0.087
Education level (primary school = 1)	0.073	−0.039
Education level (junior high school = 2)	0.040	0.041
Education level (vocational/high school = 3)	0.018	0.003
Education level (college or higher = 4)	−0.131	−0.005
Occupation (unemployed = 0, employed = 1)	−0.216	0.022

Absolute SMD < 0.1 indicates balance.

### 3.3 Interaction between region and maternal iodine nutrition on neonatal growth parameters

The interaction effects between region and maternal iodine nutrition were examined using likelihood ratio tests (Table 3). Significant interactions were observed between maternal serum iodine and neonatal birth length (*p* = 0.044), random urinary iodine and neonatal birth length (*p* = 0.004), as well as maternal random urinary iodine and neonatal birth weight (*p* = 0.016). These findings suggest regional heterogeneity in the influence of maternal iodine nutrition on neonatal growth parameters.

**Table 3:** Results of interaction analysis between geographic region and serum iodine.

Exposure factor	Outcome measure	χ^2^-value	df	*P*-value
Serum iodine	Birth length	18.527	1.4	**0.044**
	Birth weight	488292.034	1.2	0.213
Random urinary iodine	Birth length	36.545	1.6	**0.004**
	Birth weight	1921807.600	1.5	**0.016**

Likelihood ratio tests were performed by comparing the model without interaction terms (region + s(exposure, *k* = 4)) and the model with interaction terms (region + s(exposure, by = region, *k* = 4)). Bold text indicates statistical significance.

### 3.4 Differences in nonlinear effects of iodine nutrition levels on neonatal growth between rural/agro-pastoral and urban pregnant women

**Figure 2:**
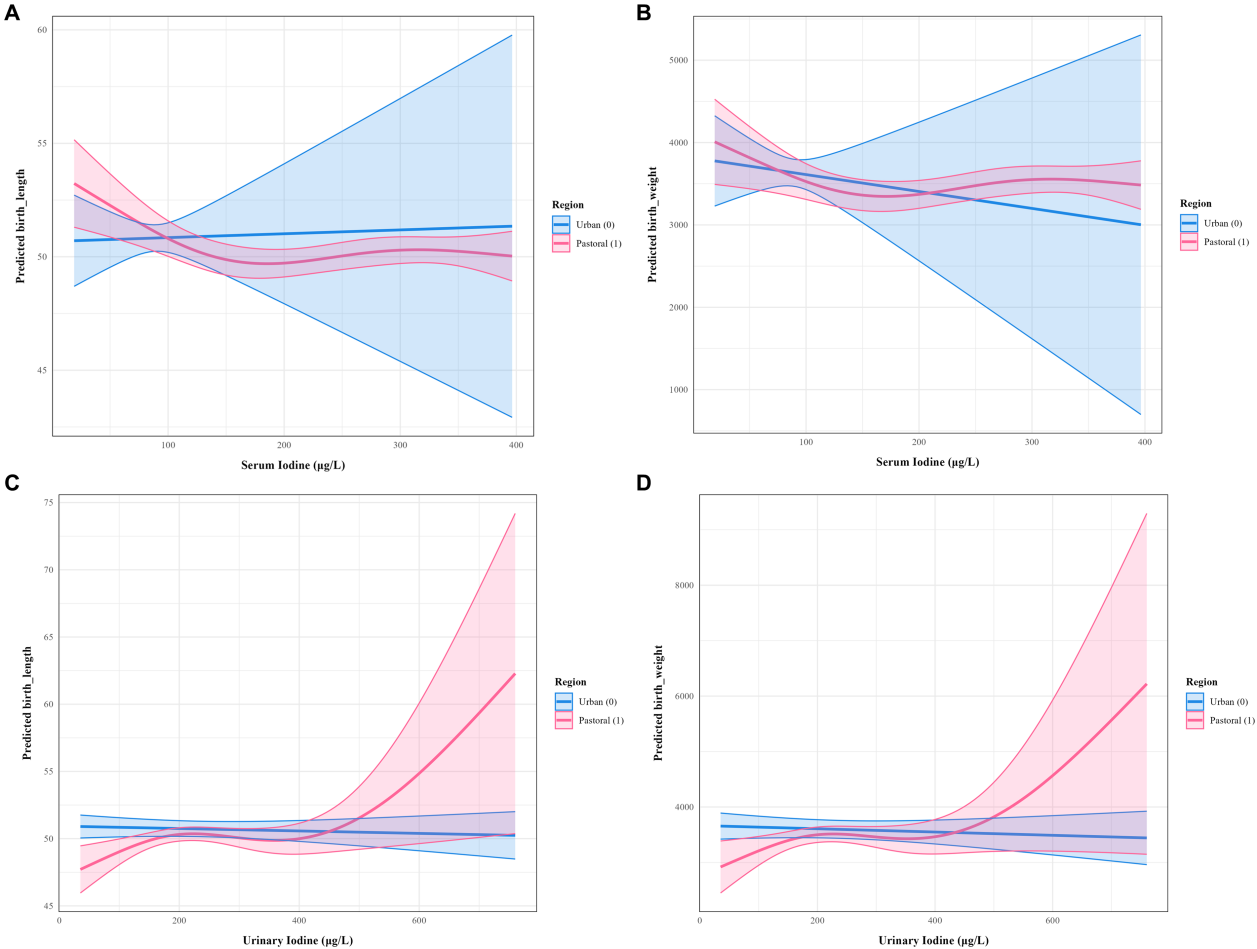
Nonlinear associations between maternal iodine nutrition levels and neonatal growth indicators in rural/agro-pastoral and urban areas. **(A)** Serum iodine versus body length; **(B)** serum iodine versus body weight; **(C)** random urinary iodine versus body length; **(D)** random urinary iodine versus body weight.

The results in Table 4 demonstrate that in rural/agro-pastoral areas, maternal serum iodine and neonatal birth length (*p* = 0.017), as well as urinary iodine and neonatal birth length (*p* = 0.030), exhibit statistically significant nonlinear associations. In contrast, iodine nutrition levels in urban pregnant women mostly show linear or no associations with neonatal growth parameters. Figure 2 visually illustrates the differences in nonlinear relationships between iodine nutrition levels and neonatal growth indicators in rural/agro-pastoral versus urban areas. The fitted curves for rural/agro-pastoral regions display nonlinear patterns, whereas those for urban areas are nearly flat.

**Table 4:** Analysis of nonlinear relationships between iodine levels and neonatal growth parameters.

Exposure factor	Outcome variable	Region	EDF	*F*-value	*P*-value
Serum iodine	Birth length	1	2.673	3.871	**0.017**
		0	1.000	0.015	0.903
	Birth weight	1	2.600	2.702	0.101
		0	1.000	0.290	0.591
Random urinary iodine	Birth length	1	2.671	2.699	**0.030**
		0	1.000	0.313	0.576
	Birth weight	1	2.568	1.895	0.085
		0	1.002	0.448	0.506

Urban = 0, Rural/Agro-pastoral = 1. Bold text indicates statistical significance.

### 3.5 Optimal exposure range of iodine nutrition levels in pregnant women from rural/agro-pastoral areas and neonatal body length

**Figure 3:**
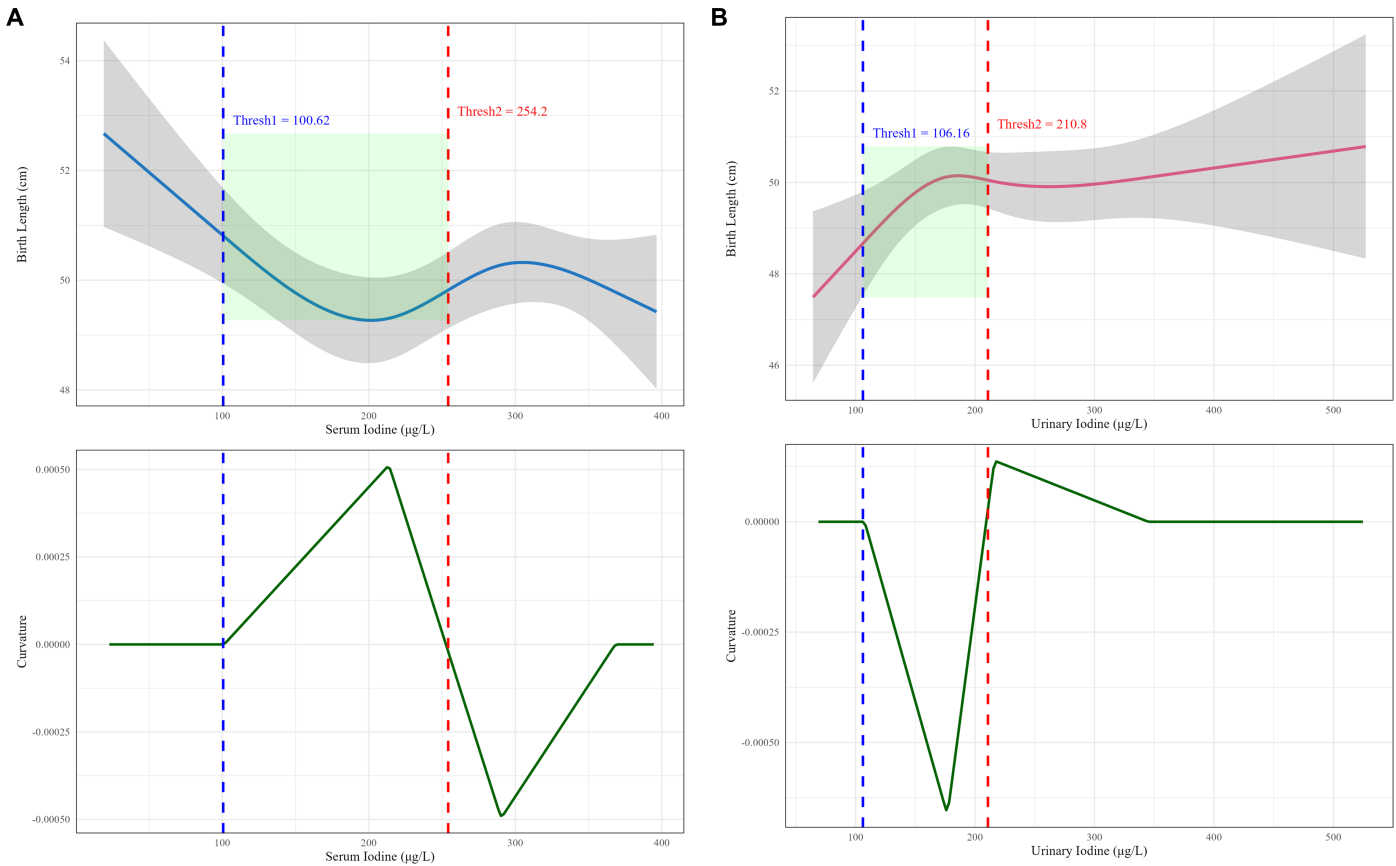
Dose-effect inflection points of maternal iodine nutrition levels and neonatal body length in rural/agro-pastoral regions. **(A)** The dose-effect inflection point between serum iodine and neonatal body length; **(B)** the dose-effect inflection point between random urinary iodine and neonatal body length.

This study identified a definitive dose–response inflection point between iodine nutrition levels in pregnant women from rural/agro-pastoral areas and neonatal body length (Figure 3). When maternal serum iodine levels ranged from 100.62 to 254.20 μg/L, the dose–response curve exhibited a “U”-shaped pattern with the narrowest confidence interval. The curvature reached zero at serum iodine concentrations of 100.62 μg/L and 254.20 μg/L, indicating that this range represents the optimal exposure window for maternal serum iodine to promote neonatal body length development. Similarly, when random urinary iodine levels in pregnant women ranged from 106.16 to 210.80 μg/L, the curve displayed an inverted “U”-shaped pattern with relatively stable confidence intervals. The curvature also approached zero at urinary iodine concentrations of 106.16 μg/L and 210.80 μg/L, suggesting that this interval constitutes the optimal range for maternal urinary iodine to enhance neonatal body length development.

## 4 Discussion

This study untangled the complexity of iodine nutrition effects under urban–rural disparity by analyzing iodine nutritional status and neonatal growth parameters between urban pregnant women in Urumqi and rural/agro-pastoral pregnant women in Ili Kazakh Autonomous Prefecture. The findings revealed significant differences in serum iodine levels between urban and rural/agro-pastoral pregnant women in Xinjiang. The median serum iodine concentration in urban pregnant women was 84.44 (75.50, 97.10) μg/L, significantly lower than that in rural/agro-pastoral pregnant women [246.41 (186.33, 322.54) μg/L]. However, no statistically significant difference was observed in random urinary iodine levels between the two groups. These findings may be related to the fact that most urban pregnant women were in their first trimester, during which the fetal thyroid gland is not fully developed, and maternal serum iodine is primarily utilized for maternal metabolism and placental transfer (28). In contrast, during the second trimester, fetal thyroid function gradually matures, allowing the mother to adapt by regulating her own iodine reserves to meet fetal demands (29–31). However, the underlying reasons for this heterogeneity should be interpreted within the context of the distinct dietary cultures and environmental backgrounds of the two regions (32). In this study, the higher iodine levels observed among pregnant women in the rural/agro-pastoral area were not driven by socioeconomic advantages or seafood consumption, but rather by a unique exposure pattern shaped by the combination of low water iodine levels and traditional ethnic dietary practices (33). The study area, the Ili rural/agro-pastoral region, is characterized by low water iodine content and a naturally low environmental iodine baseline. Local residents have long adhered to a dietary pattern—represented by traditional Kazakh cuisine—that relies heavily on iodized salt (17). Pregnant women in the rural/agro-pastoral routinely consume salty milk tea prepared with iodized salt, and iodized salt is also widely used in the processing and cooking of dairy products and meat. This dietary structure provides a consistent, stable, and relatively high dose of iodine intake, leading to significantly elevated iodine reserves that approach or even exceed the upper limit of the adequate range (34). In comparison, urban pregnant women have more diverse dietary patterns with a higher proportion of processed foods, resulting in relatively unstable iodine sources and suboptimal iodine nutrition. However, this study did not investigate the specific details of iodine intake in the two regions, and urinary iodine measurements were not corrected for creatinine concentration, which may affect the accuracy of iodine excretion assessment (35). Future studies should optimize methodology by implementing creatinine correction or 24-h total urinary iodine measurement, combined with dietary surveys, to comprehensively elucidate the iodine nutritional characteristics of pregnant women in both urban and rural areas.

After inverse probability weighting, the average weights of urban and rural groups approached 1, providing a reliable data foundation for subsequent investigation of the regional and maternal iodine nutrition effects on neonatal growth indicators. The results demonstrated significant interactions between maternal serum iodine and neonatal birth length, random urinary iodine and neonatal birth length, as well as maternal random urinary iodine and neonatal birth weight, indicating regional heterogeneity in the impact of maternal iodine nutrition on neonatal growth parameters. Nonlinear effect analysis further untangled significant nonlinear relationships between serum iodine/random urinary iodine and neonatal birth length among pregnant women in pastoral regions, whereas urban maternal iodine nutrition levels mostly exhibited linear associations or no associations with neonatal growth indicators. The limited sample size of urban pregnant women may have introduced interference in detecting association effects, potentially masking weak or nonlinear relationships (36). This heterogeneity may stem from multidimensional factors including urban–rural disparities in dietary patterns, lifestyle habits, healthcare resource allocation, and genetic backgrounds, leading to differential effects of the same iodine nutrition level on neonatal growth across regions (37–40). Due to long-term and stable high iodine intake, pregnant women in the pastoral areas of Xinjiang have extremely sufficient iodine reserves, which may even approach or exceed the upper limit of the appropriate range. Under these circumstances, iodine exhibits a significant nonlinear relationship with neonatal growth. When iodine levels are too low, they are insufficient to support fetal thyroid hormone synthesis and normal growth and development. Conversely, persistently excessive intake may trigger the Wolff–Chaikoff effect due to iodine overload, inhibiting maternal and fetal thyroid function and restricting neonatal growth (41, 42). In contrast, urban pregnant women are in a relatively lower iodine nutritional status. Their iodine intake may only be at a threshold level that barely meets daily metabolic demands. Thus, iodine nutrition is no longer a major limiting factor for neonatal growth, resulting in a weak linear association or no statistically significant relationship (15). This discrepancy highlights the regional and cultural dependency of iodine’s nutritional effects, suggesting that public health interventions should go beyond purely economic or geographic perspectives and delve into the profound influence of local dietary practices on nutrient exposure and health outcomes. Therefore, differentiated iodine nutrition intervention strategies should be formulated for urban and rural areas to achieve precise regulation of iodine nutrition during pregnancy and promote healthy neonatal growth.

This study found that when serum iodine levels in pregnant women from Xinjiang’s agricultural and pastoral areas ranged between 100.62 ~ 254.20 μg/L and random urinary iodine concentrations fell within 106.16 ~ 210.80 μg/L, an optimal dose–response relationship with neonatal length was observed. These findings align with previously recommended ranges for adequate iodine intake during pregnancy while better reflecting the actual exposure characteristics of agricultural and pastoral populations (43–45). This study identified an optimal iodine range that is consistent with the findings of a prospective maternal–infant cohort study conducted in Huizhou, Southern China. That study reported a U-shaped relationship between maternal serum iodine concentration in early pregnancy and the risk of small for gestational age (SGA). The risk of SGA began to increase when serum iodine levels exceeded 94 μg/L, suggesting that excessive iodine may restrict fetal growth (46). Another investigation examined the long-term effects of maternal urinary iodine concentration on child growth and neurodevelopment, revealing a nonlinear association between prenatal urinary iodine levels and physical development in children. Both insufficient and excessive iodine exposure were found to have potential adverse effects (47). A meta-analysis summarizing the relationship between maternal iodine status and neonatal outcomes also observed nonlinear effects, indicating that both iodine deficiency and excess were associated with abnormal neonatal anthropometric measures. These findings support the U-shaped curve identified in the present study, although the meta-analysis highlighted that the optimal iodine range may vary across different populations (48). The U-shaped and inverted U-shaped curves demonstrate that the impact of iodine nutrition on neonatal growth does not follow a simple linear relationship (5, 49). When levels fall below the lower threshold, iodine deficiency may impair thyroid hormone synthesis and consequently restrict fetal skeletal growth (50, 51). Conversely, exceeding the upper limit may trigger thyroid autoimmunity or metabolic disorders of thyroid hormones, similarly exerting negative effects on fetal development (52, 53). These results suggest that iodine supplementation strategies in agricultural and pastoral regions should avoid indiscriminate practices and maintain maternal iodine nutrition within this optimal range to effectively promote healthy neonatal growth. However, several limitations should be acknowledged. First, the study did not account for dynamic changes in fetal iodine requirements across different gestational periods, which may introduce temporal biases in the defined optimal range (54). Second, potential confounding factors—including maternal thyroid hormone levels during pregnancy, genetic background, and gestational BMI changes—were not fully controlled for in the analysis (55, 56). These factors may independently or synergistically influence neonatal linear growth, thereby interfering with accurate assessment of iodine nutrition effects. Third, using neonatal length as the sole outcome measure without comprehensive evaluation of other critical dimensions limits the ability to fully characterize iodine’s impact on fetal health (6, 57). Future studies should employ prospective birth cohort designs incorporating dynamic multi-parameter monitoring and rigorous adjustment for confounders to validate and refine this optimal exposure range, thereby providing more robust scientific evidence for precision-based iodine nutrition interventions.

This study untangles the regional heterogeneity in the impact of maternal iodine nutrition levels on neonatal growth between urban and rural/agro-pastoral areas in Xinjiang. Significant disparities in serum iodine levels among pregnant women were observed between urban and rural regions, with region-specific effects of iodine nutrition. Notably, a definitive dose–response inflection point was identified between maternal iodine nutrition status and neonatal body length in rural/agro-pastoral areas. The optimal exposure ranges of maternal serum iodine and random urinary iodine for promoting neonatal linear growth were established, providing a scientific basis for iodine nutrition interventions during pregnancy. Although this study has revealed several profound findings, it is essential to acknowledge several methodological limitations that directly affect the interpretation of the results and define the scope of the conclusions. First, the imbalance in sample size is a central issue. In particular, the urban subgroup had a relatively small sample size, which not only reduced statistical power but also likely hindered the detection of genuinely weak associations or more complex nonlinear patterns that may exist within this population. This limitation implies that the reported linear or null associations in the urban subgroup should be considered preliminary and interpreted with caution. Second, the failure to systematically collect dietary iodine intake data constitutes a significant omission. This prevented precise quantification of total iodine intake and differentiation of contributions from various dietary sources. Furthermore, the lack of creatinine adjustment for random urinary iodine concentrations may have introduced measurement variability due to individual differences, thereby compromising the accuracy of recent iodine intake assessment. The findings of this study, particularly the adequate range identified in the rural/agro-pastoral areas, may only be applicable to specific populations in Xinjiang with similar dietary and cultural backgrounds. Extrapolation of these results to other regions or ethnic groups must be undertaken with extreme caution. Future research should aim to address these shortcomings by expanding sample sizes, incorporating detailed dietary assessments along with creatinine-adjusted urinary iodine analysis, and adopting prospective designs that include multidimensional child health outcomes. Such efforts would yield more robust and comprehensive evidence.

## 5 Conclusion

This study investigated the differences in the impact of maternal iodine nutrition levels on neonatal growth between urban and rural areas in Xinjiang. The results suggest that the association between iodine nutrition and neonatal growth is region-specific, with a nonlinear relationship and an optimal range observed particularly between iodine levels and neonatal body length in the rural/agro-pastoral populations. These findings provide preliminary evidence for developing region-specific interventional strategies regarding maternal iodine nutrition. However, this study has several limitations, including sample size, dietary information collection, and urinary iodine correction. Therefore, the conclusions should be further validated in larger and more rigorously designed studies. Future research should be conducted in other regions of China with uneven iodine nutrition status to compare the relationships between iodine exposure patterns and health outcomes, thereby providing a scientific basis for establishing more generalizable and precise nutritional intervention strategies.

## Data Availability

The datasets presented in this article are not readily available because the data involved in this study are not publicly available due to privacy but are available from the authors on reasonable request. Requests to access the datasets should be directed to Chenchen Wang, 357935099@qq.com.
